# Development & application of a wearable non-differential calorimeter for skin heat transfer analysis

**DOI:** 10.1371/journal.pone.0334062

**Published:** 2025-10-17

**Authors:** Pedro Jesús Rodríguez de Rivera, Miriam Rodríguez de Rivera, Fabiola Socorro, Manuel Rodríguez de Rivera

**Affiliations:** 1 Department of Physics. Campus de Tafira, Universidad de Las Palmas de Gran Canaria (ULPGC), Las Palmas de Gran Canaria, Spain; 2 Cardiology Service. Hospital Universitario Marqués de Valdecilla, Santander, Spain; Himeji Dokkyo University, JAPAN

## Abstract

The thermal properties of human skin are of great interest for understanding local and global body heat loss, various physiological responses or even skin injuries. This study presents a wearable, non-invasive skin calorimeter designed for *in vivo* measurement of skin heat flux, heat capacity, and thermal resistance. The device, based on the principle of non-differential heat conduction calorimetry, consists of a programmable thermostat, a heat flux sensor and a Peltier cooling system. To operate the device, we propose and calibrate a calorimetric thermal model that includes the skin. This new model approach allows to estimate the core temperature of the tissue where the measurement is performed. Experimental validation of the device was carried out on localized skin areas, both at rest and during moderate physical activity. This skin calorimeter allows determination of thermal properties in different skin regions, with an accuracy of ± 2 mW for the heat flux, ± 1 K/W for the thermal resistance, and ± 0.05 J/K for the heat capacity, for a 2 × 2 cm² skin region. The results confirm the applicability of these devices in sports medicine, thermoregulation studies, and medical diagnostics. This work also includes simulations of the calorimeter’s operation, which help to define its operating range and to study the interaction between the device and the human skin.

## 1. Introduction

The thermal properties of human skin have attracted growing interest, due to their relevance in thermoregulation, heat loss, and their association with various skin conditions. As early as 1977, ML. Cohen [1] highlighted the need for further research in this area, citing a lack of published data. In recent years, some emerging databases have begun to compile data [2], helping to address this gap. While these databases compile in vivo parameters such as metabolic rate and blood perfusion, some thermal properties like heat capacity and thermal conductivity are consistently tabulated from in vitro or ex vivo measurements, showing negligible temperature dependence. Since water content is essential for these properties but highly variable across individuals and conditions, in vivo measurements are necessary to capture this variability. In vivo thermal analysis of skin, an emerging area of research, has proven useful across several fields. In medicine, for instance, it has been applied to melanoma detection [3,4], monitoring the evolution of skin lesions [5], and even to track the effects of anesthesia [6]. Some experiments have been also performed in the field of sports medicine, for evaluating the influence of clothing and environmental conditions [7–9], as well as the effects of aging and fitness on thermoregulation during exercise in hot environments [10]. Similar work has examined neural factors underlying age-related impairments in reflex cutaneous vasodilation [11].

On the other hand, measuring the heat capacity and the thermal resistance of the skin is essential for simulating its thermal behavior under various conditions; for instance, when it is affected by certain pathologies [12] or to model the thermal dissipation of a subject under different environmental and clothing scenarios [13]. These simulations are mainly based on solving the Fourier equation of conductive heat transfer [14]. This equation, solved with the finite element method, allows the incorporation of additional physiological terms such as blood perfusion and cells with high thermal activity [15,16]. *In vivo* and *in vitro* determination of skin heat capacity and thermal resistance is of great interest, since simulations using empirically derived values are more representative of living tissue. *In vitro* measurements of skin heat capacity are typically obtained using differential scanning calorimetry [2], which involves extracting a tissue sample for analysis. In contrast, *in vivo* measurement of skin thermal properties is an underdeveloped field. Some recent technologies allow the assessment of skin thermal resistance [17–19], heat capacity [20–22] and thermal diffusivity [19,23]. However, recent studies have shown the dependence of these results on the thermal penetration depth and other factors, making comparisons difficult [24,25]. The main challenge lies in understanding the heat transfer phenomena. Thermal properties directly depend on the heat flux, which is influenced by convection and radiation effects. If these factors are not controlled, the uncertainty becomes significant. Consequently, all research efforts in this area are of great interest for the advancement of the field. While temperature measurements are generally precise, the determination of local heat flux or energy expenditure remains notoriously inaccurate. For this reason, this work includes a detailed analysis of the heat flux pathways from the skin to the sensor’s thermostat and to the ambient. Our instrument minimizes convection and radiation phenomena. In all current technologies for *in vivo* measurement of skin thermal properties, the procedure generally follows a common principle: a thermal excitation, typically a slight heating or cooling, is applied to the skin. Then, the analysis of the transient response of the thermal sensor allows to determine the heat capacity and/or the thermal resistance of the skin. Since the thermal properties of the skin are directly linked to heat loss, accurate heat flux assessment is essential. One application of special interest is the monitoring of skin lesions [5]. For instance, burn depth estimation can be valuable in perioperative thermal management to determine when skin thermal parameters normalize. Heat flux assessment is also useful for localized muscle performance analysis [7,10], and for the validation of wearable thermal sensors, since many devices share similar designs.

We have developed a skin calorimeter capable of characterizing the thermal properties of a localized 2 x 2 cm^2^ skin area. This instrument belongs to the category of non-differential conduction calorimeters according to Hansen’s classification [26]. Typically, conduction calorimeters work in differential setup, using two identical cells. In one of them the thermal process under study takes place, and the other cell (empty) is used as a reference. This setup ensures that variations in the thermostat or ambient temperature do not affect the measurement. However, skin thermal sensors are inherently non-differential, meaning that both ambient and thermostat temperature fluctuations influence the measured signals. Therefore, a full calibration of these skin calorimeters is required to determine the power transmitted through the calorimeter under any ambient or thermostat temperature.

The calorimeter was initially designed to measure skin heat flux; however, its progressive development has recently enabled the measurement of both skin heat capacity [25] and thermal resistance [24] under resting conditions. In recent months, we have worked on calorimetric models and procedures to measure these quantities during physical activity [27]. In this work, we present a calorimetric model and a calibration procedure that allow the device to operate without baseline correction, which substantially improves the potential applications of the instrument, particularly in wearable technologies. In addition, this enables the characterization of the heat fluxes through each component of the device, and also allows the estimation of the internal core temperature of the specific body area where the measurement is performed.

In this paper, we first describe the experimental system and its operating model. This is followed by a detailed study of the calibration process. Applications of the calorimeter on human skin at rest and during exercise are shown, and finally, several simulations are presented, in order to illustrate the operating domain of the device and validate the model equations proposed.

## 2. Experimental system and calorimetric model

**Fig 1:**
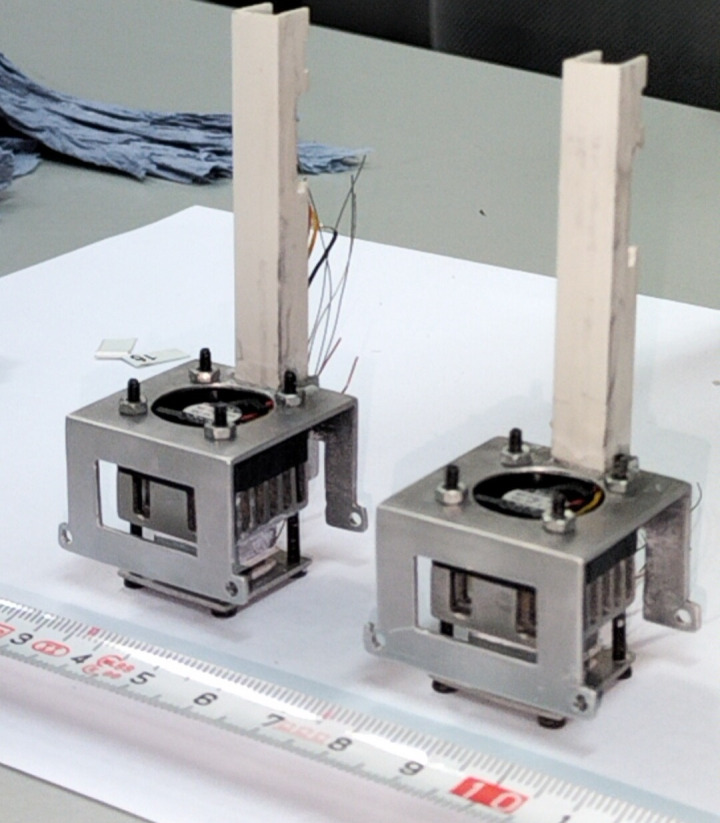
Skin calorimeters during assembly. Skin calorimeters during the assembly process, prior to attaching the lateral thermal insulation, applying the fastening system, and connecting the wires to the sensors.

Two prototypes of the skin calorimeter, each with a measuring area of 2 x 2 cm^2^, have been constructed [27]. The core component is a measuring thermopile (ET12–65-F2A-1312-11-W2.25, Laird Thermal Systems [28]), which is placed between an aluminum measuring plate (20 x 20 x 1 mm), which contacts the skin or the sample under study, and an aluminum thermostat (14 x 14 x 4 mm) located on the opposite side of the thermopile. The thermostat regulates the temperature of the calorimeter’s thermal focus and contains a temperature sensor (PT100GO1020HG, Omega Engineering [29]) and a heating resistor. A Peltier system is used to cool the thermostat. It consists of a thermopile identical to the measuring one, a heatsink, and a fan. The measuring thermopile, the thermostat, and the cooling thermopile are laterally insulated with expanded polystyrene, and covered with a thin reflective aluminum foil. A calibration base has also been constructed, incorporating an electrical resistor. The resistors in both the thermostat and the calibration base were fabricated using Teflon-insulated constantan wire (TFCC-005–50 by Omega Engineering [29]). **Fig 1** shows the skin calorimeters during the assembly process, prior to attaching the lateral thermal insulation, the fastening system, and the wire connection to the sensors.

To interpret the calorimeter’s behavior, its operation is described using a simplified mathematical representation. The operating model of the calorimeter is based on RC models, which are commonly used in calorimetry [30]. These models represent the experimental system as a network of discrete domains represented by its heat capacity, interconnected through thermal couplings (thermal conductances). In these kind of models, each domain is assumed to have infinite thermal conductivity, implying a uniform temperature throughout its volume. The number of domains is determined through preliminary calibration measurements, performed to identify the minimum number of poles required for a transfer function to adequately reproduce the calorimetric signal measured by the thermopile in response to the power dissipated in the calibration resistor. This procedure is common in instrumentation to ensure that the model accurately represents the system’s dynamic behavior. For the calorimeters used in this study, we verified that a second order Transfer Function is enough to accurately describe the behavior of the system. Therefore, the calorimeter is modeled into two thermal domains: the first one includes the heat source, the measuring plate, and the portion of the thermopile in contact with the plate. The second one includes the thermostat and the portion of the thermopile in contact with it. Under this configuration, the system of equations of the model is defined as follows:


$$\begin{array}{c} W_{1}=C_{1}\frac{dT_{1}}{dt}+P_{1}(T_{1}-T_{01})+P_{12}(T_{1}-T_{2})\\ W_{2}=C_{2}\frac{dT_{2}}{dt}+P_{2}(T_{2}-T_{02})+P_{12}(T_{2}-T_{1}) \end{array}$$ (1)

Each domain is characterized by its heat capacity (*C*_*1*_ and *C*_*2*_), the power generated inside it (*W*_*1*_ and *W*_*2*_), and its temperature (*T*_*1*_ and *T*_*2*_). The thermal conductance *P*_*12*_ corresponds to the measuring thermopile that connects the two domains. *P*_*1*_ represents the thermal conductance between the first domain and the external environment, which is at a temperature *T*_*01*_, while *P*_*2*_ corresponds to the thermal conductance between the thermostat and the cooling system, which is at a temperature *T*_*02*_. The calorimetric signal provided by the measuring thermopile is linearly related to the temperature difference between domains, by the Seebeck coefficient *k*: *y* = *k* (*T*_*1*_ – *T*_*2*_). By subtracting *T*_*1*_ and substituting in Eq. 1, we obtain the system of equations of the calorimetric model:


$$\begin{array}{c} W_{1}=\frac{C_{1}}{k}\frac{dy}{dt}+C_{1}\frac{dT_{2}}{dt}+\frac{P_{1}+P_{12}}{k}y+P_{1}(T_{2}-T_{01})\\ W_{2}=C_{2}\frac{dT_{2}}{dt}-\frac{P_{12}}{k}y+P_{2}(T_{2}-T_{02}) \end{array}$$ (2)

This model relates the output signals, the calorimetric signal *y* (t) and the thermostat temperature *T*_*2*_ (t), to the input signals: the power dissipated by the skin or the calibration base *W*_*1*_ (t), and the thermostat power *W*_*2*_ (t). In addition to the relationships between power and temperature, it is necessary to consider the influence of the thermostat’s cooling system. Due to the Peltier effect, one side of the cooling module is cooled, while the other side is heated, and must be actively cooled by the heatsink and fan. As a result, the external temperatures *T*_*01*_ and *T*_*02*_ deviate from the ambient temperature *T*_*room*_. These effects can be expressed through the following linear relationships, which depend on the cooling system supply current *I*_*pel*_:


$$T_{01}=T_{0}+T_{room}+\alpha I_{pel}\;\;\;\;\;;\;\;\;\;\;T_{02}=T_{0}+T_{room}+\beta I_{pel}$$ (3)

We experimentally verified that these relationships are linear for *I*_*pel*_ < 250 mA. The parameters *α*, *β* and *T*_*0*_ were determined with the calorimeter placed on the calibration base. In this configuration, the power *W*_*1*_ corresponds to the power dissipated in the calibration resistor. However, when the calorimeter is placed on the skin, *W*_*1*_ represents the heat flux transferred by conduction from the skin to the calorimeter. In this case, *T*_*0*_ is typically higher, and is therefore determined for each measurement. However, parameters *α* and *β* are invariant.

The power *W*_*2*_ is regulated by a PID controller to maintain the thermostat at the programmed temperature *T*_*2*_. The calorimetric signal *y* and the temperatures *T*_*room*_ and *T*_*2*_ are acquired using a data acquisition system (Keysight 34970A with 34901A multiplexer module). The current *I*_*pel*_ and the powers *W*_*1*_ and *W*_*2*_ of the heating resistors are supplied by a programmable triple-output power supply (Keysight E3631A). The acquisition program, written in C++, controls all the instruments via the GPIB interface with a sampling period of Δt = 1 s.

## 3. Calibration

**Fig 2:**
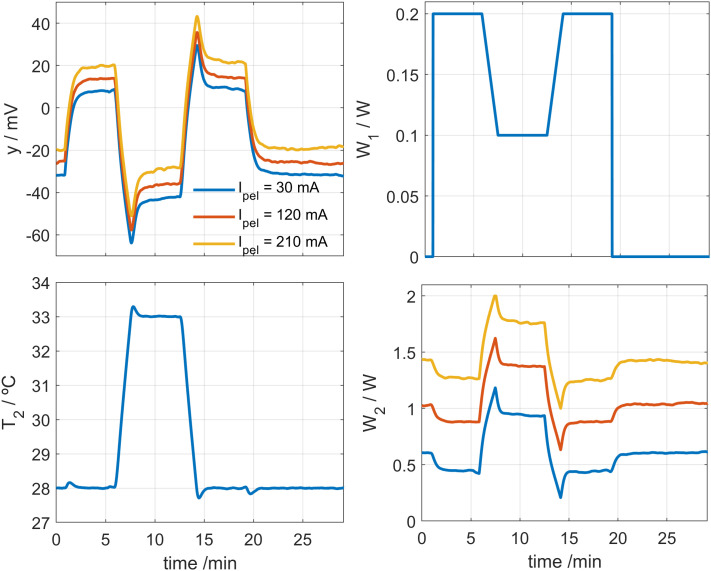
Calibration measurement. Calibration measurement performed at a room temperature of 21.2°C, for three different cooling thermopile currents (*I*_*pel*_ values of 30, 120 and 210 mA). Thermostat temperature *T*_*2*_ varied from 28 to 33°C. Calorimetric signal *y*, calibration base power *W*_*1*_, and thermostat power *W*_*2*_ are shown.

Calibration is carried out by placing the calorimeter on a small aluminum block that contains a heating resistor, located in the calibration base. To identify the calorimetric model, we performed experiments by modifying three key parameters: the supply current of the cooling thermopile *I*_*pel*_, the thermostat temperature *T*_*2*_, and the power dissipated in the calibration base *W*_*1*_. An example of these calibration measurements is shown in **Fig 2**. In these measurements, the thermostat temperature was initially programmed at *T*_*2*_ = 28°C. Once steady state was reached, it was increased to *T*_*2*_ = 33°C, held for 5 minutes, and then reduced back to *T*_*2*_ = 28°C. The heating and cooling rate was 3 K/min. Simultaneously, a dissipation of *W*_*1*_ = 0.2 W was applied, which was reduced to 0.1 W during the thermostat temperature increase, then returned to 0.2 W and finally set to zero. **Fig 2** shows measurements for *I*_*pel*_ values of 30 mA, 120 mA and 210 mA, covering a wide margin of the operating domain. The linearity of the device is demonstrated, as all the determined parameters remain constant. In other words, when these parameters are introduced into the model, the calorimeter performance is accurately reproduced for all calibration measurements. In these tests, the heat flux (*W*_*1*_), the thermostat power and temperature (*W*_*2*_ and *T*_*2*_), and the Peltier current (*I*_*pel*_) were deliberately varied to cover a broad operating range.

The complete calibration of the calorimeters is performed in two phases. In the first phase, the baselines of all experimental curves are corrected, and the model parameters are determined: the heat capacities *C*_*1*_ and *C*_*2*_, the thermal conductances *P*_*1*_, *P*_*2*_ and *P*_*12*_, and the Seebeck coefficient *k*. In the second phase, baseline correction is not applied, and the parameters *α*, *β* and *T*_*0*_ (Eq. 3) are determined for each value of the cooling thermopile supply current. These parameters characterize the influence of the cooling system. Sections 3.1 and 3.2 describe these two steps of the calibration process in detail.

### 3.1. Determination of RC model parameters

During the calibration measurements, ambient temperature variations are negligible, and the cooling thermopile current (*I*_*pel*_) is constant. As a result, the initial and final states are nearly identical, and, according to Eq. 3, the temperatures *T*_*01*_ and *T*_*02*_ can be considered constant. Under these conditions, baseline correction is possible, and the model can be expressed as follows, where the input are Δ*W*_*1*_ and Δ*W*_*2*_, and the outputs Δ*y* and Δ*T*_*2*_:


$$\begin{array}{c} \Delta W_{1}=\frac{C_{1}}{k}\frac{d\Delta y}{dt}+C_{1}\frac{d\Delta T_{2}}{dt}+\frac{P_{1}+P_{12}}{k}\Delta y+P_{1}\Delta T_{2}\;\\ \Delta W_{2}=C_{2}\frac{d\Delta T_{2}}{dt}-\frac{P_{12}}{k}\Delta y+P_{2}\Delta T_{2} \end{array}$$ (4)

To determine the model parameters, we use an iterative algorithm that reconstructs the output signals from the input signals and the model parameters, and compares these reconstructed signals with the experimental ones. At each iteration, the algorithm calculates new values of the model parameters by minimizing an error criterion, which is the root mean square error (RMSE) between the experimental (exp) and calculated (cal) signals:


$$\varepsilon =\frac{\text{1}}{np}\sqrt{\sum_{i=1}^{np}{\left({T_{2}}_{\exp}(i)-T_{2cal}(i)\right)}^{2}}+\frac{\text{1}}{np}\sqrt{\sum_{i=1}^{np}{\left(y_{\exp}(i)-y_{cal}(i)\right)}^{2}}=\varepsilon_{T_{2}}+\varepsilon_{y}$$ (5)

In this expression, ε_*T2*_ and ε_*y*_ are the RMSE values of the thermostat temperature and the calorimetric signal, and *np* is the number of data points. The fitting was performed using Matlab’s fminsearch function [31], which is based on the Nelder-Mead minimization algorithm [32,33]. The model parameters obtained for each calorimeter, *S1* and *S2*, through this process are summarized in **Table 1**. Among these parameters, *C*_*1*_ is the only variable parameter, as it depends on the heat capacity of the sample. All other parameters remain constant. The maximum errors in the fits are 18.9 µV and 5.9 mK.

**Table 1:** RC model parameters.

Calorimeter	*C*_*1*_	*C*_*2*_	*P*_*1*_	*P*_*2*_	*P*_*12*_	*k*
S1	4.02 ± 0.09	3.80 ± 0.20	0.029 ± 0.002	0.057 ± 0.005	0.092 ± 0.008	23.7 ± 1.1
S2	3.91 ± 0.09	3.70 ± 0.30	0.029 ± 0.002	0.055 ± 0.005	0.089 ± 0.009	23.0 ± 1.4
unit	J/K	J/K	W/K	W/K	W/K	mV/K

*RC* model parameters obtained using the calibration algorithm. Heat capacities (*C*_*1*_ and *C*_*2*_), thermal conductances (*P*_*1*_, *P*_*2*_ and *P*_*12*_), and Seebeck coefficient (*k*) are reported as mean ± standard deviation for both calorimeters, *S1* and *S2*. Units are indicated below each parameter.

### 3.2. Evaluation of cooling system effects

**Fig 3:**
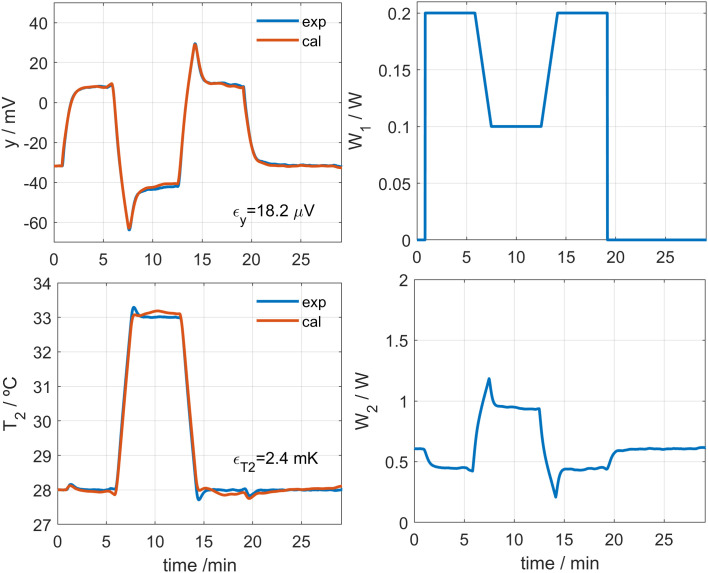
Calibration without baseline correction. Example of calibration without baseline correction. Experimental curves (blue) and model-generated curves (red) are shown: calorimetric signal (*y*), thermostat temperature (*T*_*2*_), calibration base heat flux (*W*_*1*_), and thermostat power (*W*_*2*_). The experiment was performed using the *S1* calorimeter, at a room temperature of 21.2°C and with a cooling current of *I*_*pel*_ = 30 mA. RMSE values are also shown.

**Fig 4:**
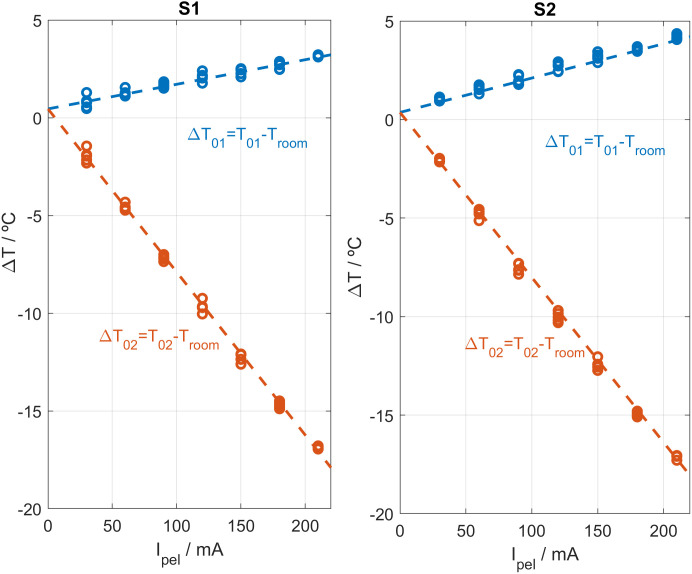
Δ*T*_*01*_ and Δ*T*_*02*_ values determination. Experimental values of Δ*T*_*01*_ = *T*_*01*_ – *T*_*room*_ (blue) and Δ*T*_*02*_ = *T*_*02*_ – *T*_*room*_ (red) as a function of the cooling thermopile supply current *I*_*pel*_. Results are shown for both skin calorimeters, *S1* and *S2*.

The process described in the previous section operates directly on changes of the input and output signals and fits adequately. However, the initial and final values of the heat flux *W*_*1*_ are lost due to the baseline correction. To calibrate a model capable of operating without baseline correction, and therefore recover the absolute values of *W*_*1*_, it is necessary to take into account the effects of the cooling system. If the parameters α, β, and *T*_*0*_ are determined from Eq. 3, the temperatures *T*_*01*_ and *T*_*02*_ can be obtained, completing the identification of the model (Eq. 2). To achieve this, a procedure similar to that described previously [31–33] is applied, but now the model equations fitted correspond to system Eq. 2 instead of Eq. 4. We start from the model parameters already obtained (Table 1) and fit Eq. 3 and Eq. 4. Fig 3 shows the fit between the experimental and the model-generated curves for a calibration measurement. Fig 4 shows the calculated values of Δ*T*_*01*_ and Δ*T*_*02*_ for each *I*_*pel*_ value. As mentioned above, the linearity of Eq. 3 is experimentally verified in the studied cases, which represent the operating range of the calorimeters.

Table 2 shows the coefficients α and β of the linear fits between Δ*T*_*01*_ *and* Δ*T*_*02*_, and the cooling current *I*_*pel*_. It is interesting to note that the temperature *T*_*0*_ was determined while the calorimeters are placed on the calibration base. When the calorimeters are applied on the skin, it is necessary to consider the warming of the environment near the device due to its proximity to the human body, which causes a *T*_*0*_ increase. Since the human body has a complex behavior [17–22], it is required to identify *T*_*0*_ for each measurement performed on the skin.

**Table 2:** Δ*T*_*01*_ and Δ*T*_*02*_ linear fit parameters.

	ΔT_*01*_ = *T*_*01*_ – *T*_*room*_ = *T*_*0*_ + α·*I*_*pel*_			Δ*T*_*02*_ = *T*_*02*_ – *T*_*room*_ = *T*_*0*_ + β·*I*_*pel*_		
Calorimeter	T0 (ºC)	α (ºC/mA)	*r*	*T*_*0*_ (ºC)	β (ºC/mA)	*r*
S1	0.45	0.0136	0.9711	0.45	−0.0835	−0.9990
S2	0.36	0.0174	0.9885	0.36	−0.0838	−0.9988

Parameters α and β of the linear fitting of Δ*T*_*01*_ and Δ*T*_*02*_ values, for different *I*_*pel*_ values (in miliamperes). The Pearson correlation coefficient (*r*) is reported in the table. The number of data points used in the fitting is *np* = 35.

## 4. Measurements in human skin

The study was performed in accordance with the Declaration of Helsinki, and was approved by the Human Experimentation Ethics Committee of the University of las Palmas de Gran Canaria, (protocol CEIH-2024-02, approved in April 2024). All participants were healthy adult volunteers and provided written informed consent prior to participation. Participants understood the study objectives, procedures and potential risks before signing the consent form. No minors were included in the study. Human participants were prospectively recruited between 15 and 30 September 2024.

### 4.1. Determination of skin heat flux and heat capacity

To estimate the skin heat capacity (*C*_*1*_), the applied thermal excitation consists of modifying the thermostat temperature (*T*_*2*_). This induces a skin heat flux (*W*_*1*_) variation. As reported in previous works [24,25], when the thermostat temperature increases, *W*_*1*_ decreases, following the relationship described by Eq. 6. By analyzing the response of the calorimeter, both *W*_*1*_ and *C*_*1*_ can be obtained. In Eq. 6, *T*_*2*_ (t) denotes the time-dependent thermostat temperature, with *T*_*2*_ (0) as its initial steady-state value and Δ*T*_*2*_ as the maximum temperature difference. The heat flux is defined as its initial steady-state (*W*_*10*_) plus the heat flux change influenced by thermostat temperature *T*_*2*_, following the expression:


$$W_{1}(t)=W_{10}+\frac{T_{2}(0)-T_{2}(t)}{\Delta T_{2}}\Delta W_{1}$$ (6)

In contrast to the Joule calibration measurements, in this case the skin heat flux follows the form defined by Eq. 6, which requires the knowledge of *W*_*10*_ and Δ*W*_*1*_. In addition, the parameters *C*_*1*_ and *T*_*0*_, which depend on the thermal state of the skin at the time of the experiment, must be calculated. We determine *C*_*1*_, *W*_*10*_, Δ*W*_*1*_ and *T*_*0*_ using the same method described in section 3: the Nelder-Mead minimization algorithm [32,33].

**Fig 5:**
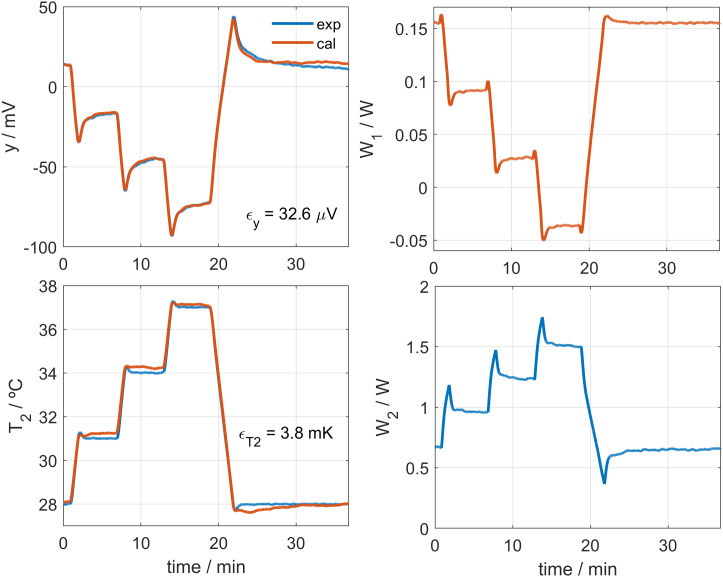
Measurement on the wrist. Experiment performed on the volar side of the left wrist of a healthy 67-year-old male subject. Experimental and calculated calorimetric signal (*y*_*exp*_ and *y*_*cal*_) and thermostat temperature (*T*_*2exp*_ and *T*_*2 cal*_), estimated skin heat flux (*W*_*1*_), and thermostat power (*W*_*2*_) are shown. RMSE values of the fits are also indicated. The measurement was performed using the calorimeter *S2*, at a room temperature of 21.2°C, with a cooling system current of *I*_*pel*_ = 100 mA.

**Fig 6:**
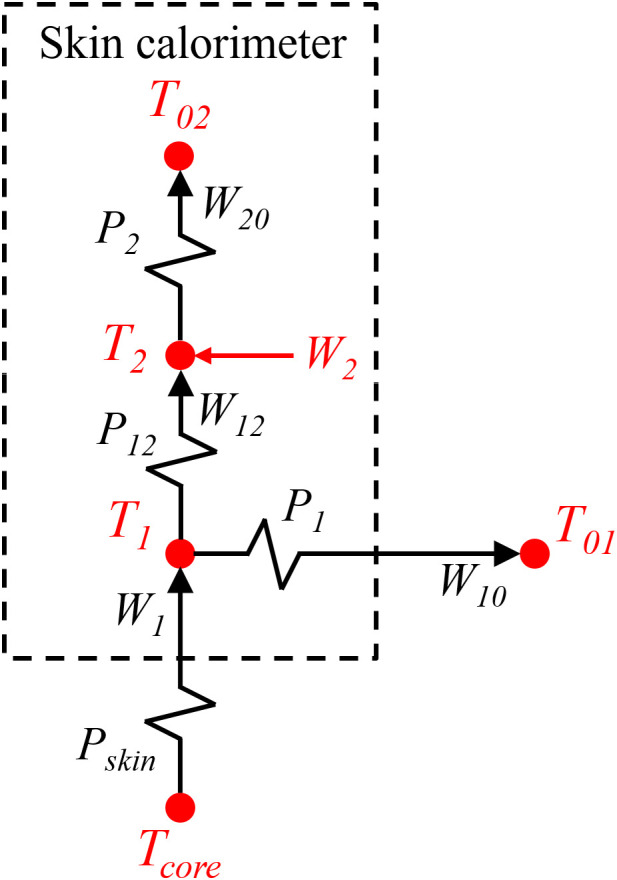
Schematic of temperatures and heat fluxes through the skin calorimeter. Diagram showing the temperatures and heat fluxes involved in heat transfer through the calorimeter. The schematic summarizes the directions of heat fluxes between the skin, the measuring and cooling thermopiles, and the environment.

In previous works, we have performed several measurements in different subjects and anatomical regions [34]. Fig 5 shows an experiment performed on the volar side of the wrist of a healthy 67-year-old male subject at rest. Both the experimental and modelled calorimetric signals (*y*_*exp*_ and *y*_*cal*_), the experimental and calculated thermostat temperatures (*T*_*2exp*_ and *T*_*2 cal*_), the estimated skin heat flux (*W*_*1*_), and the thermostat power (*W*_*2*_) are shown. In this measurement, a stepped change in the thermostat temperature was applied to verify that the determined power *W*_*1*_ is also stepwise, and that the fits for both the calorimetric signal and the thermostat temperature are acceptable. The average value of *T*_*0*_ was 2.3°C, and the heat capacity obtained was *C*_*1*_ = 4.7 J/K. Fig 6 illustrates the heat exchange pathways through the calorimeter, while Table 3 summarizes the corresponding temperatures and heat fluxes for the experiment shown in Fig 5.

**Table 3:** Temperatures and heat fluxes corresponding to the wrist measurement.

*y*	*W*_*1*_	*W*_*10*_	*W*_*12*_	*W*_*2*_	*W*_*20*_	*T*_*1*_	*T*_*01*_	*T*_*2*_	*T*_*02*_
13.2	0.155	0.104	0.051	0.669	0.720	28.6	25.0	28.0	15.0
−16.7	0.092	0.157	−0.065	0.959	0.894	30.3	25.0	31.0	15.0
−45.5	0.027	0.204	−0.177	1.235	1.058	32.0	25.0	34.0	15.0
−72.3	−0.037	0.245	−0.282	1.502	1.220	33.8	25.0	37.0	15.0
mV	W	W	W	W	W	ºC	ºC	ºC	ºC

Data of the measurement performed on the volar side of the left wrist of a healthy 67-year-old male subject at rest (**Fig 5**), using calorimeter *S2*, at a room temperature of 21.1°C, *T*_*0*_ = 2.3°C, with *I*_*pel*_ = 100 mA. The sign convention for heat fluxes (*W*_*1*_, *W*_*12*_, *W*_*10*_, and *W*_*20*_) is defined in **Fig 6**. The thermostat power dissipated (*W*_*2*_) is always positive.

At the beginning of the wrist measurement, when the thermostat stabilizes at 28°C, the system reaches a steady state in which skin heat is dissipated out of the human body (*W*_*1*_). This outgoing heat flux is split between the measuring thermopile (*W*_*12*_) and the environment (*W*_*10*_). Simultaneously, the power supplied to the cooling thermopile (*W*_*20*_) is distributed between the thermostat (*W*_*2*_) and the measuring thermopile (*W*_*12*_). As we can see, *W*_*1*_ remains positive below *T*_*2*_ = 34°C, but from 31°C onwards, the heat flux through the thermopile (*W*_*12*_) becomes negative. At 37°C, both *W*_*1*_ and *W*_*12*_ are negative. These changes in heat flux direction are consistent with the system temperatures (see Table 3).

**Fig 7:**
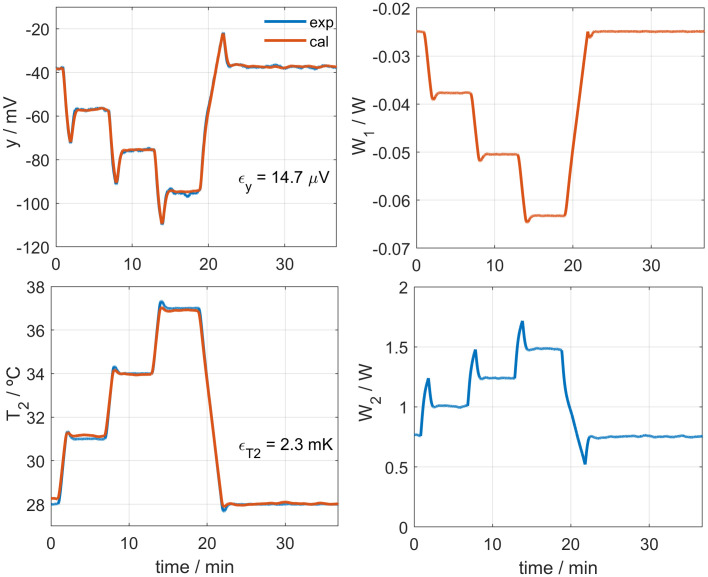
Measurement to determine the offset heat capacity. This experiment was performed to estimate the offset heat capacity. The figure displays the calorimetric signal (*y*), the thermostat temperature (*T*_*2*_), the estimated heat flux (*W*_*1*_), and the thermostat power (*W*_*2*_). Experimental data are shown in blue and modelled curves in red, with RMSE values indicated. The test was performed using calorimeter *S1*, at a room temperature of 21.2°C, with a cooling current of *I*_*pel*_ = 50 mA. The resulting offset heat capacity was *C*_*0*_ = 2.31 J/K.

To isolate the calorimeter’s own thermal response, tests were conducted with the device operating in air, without contact with the skin or the calibration base. The thermostat followed the same temperature setting as in the previous measurement. This configuration allows the estimation of the offset heat capacity (*C*_*0*_), which must be subtracted from the measured heat capacity (*C*_*1*_) to obtain the skin heat capacity: *C*_*skin*_ = *C*_*1*_ – *C*_*0*_. Fig 7 shows the fitted and experimental signals, while Table 4 report the heat flux values. The *C*_*0*_ value was 2.31 ± 0.07 J/K.

**Table 4:** Thermal data used to estimate the offset heat capacity.

*y*	*W*_*1*_	*W*_*10*_	*W*_*12*_	*W*_*2*_	*W*_*20*_	*T*_*1*_	*T*_*01*_	*T*_*2*_	*T*_*02*_
−38.3	−0.025	0.124	−0.149	0.762	0.613	26.4	22.2	28.0	17.5
−56.4	−0.038	0.182	−0.220	0.999	0.779	28.6	22.2	31.0	17.5
−75.3	−0.050	0.244	−0.294	1.240	0.946	30.8	22.2	34.0	17.5
−94.9	−0.063	0.307	−0.370	1.480	1.110	33.0	22.2	37.0	17.5
mV	W	W	W	W	W	ºC	ºC	ºC	ºC

The table presents temperature and heat fluxes values corresponding to the test shown in **Fig 7**, performed to assess the offset heat capacity *C*_*0*_.

The sensor model is consistent in the three scenarios: calibration base, measurement on skin, and measurement with no sample. The accuracy of the calculated heat flux is influenced by the fluctuations of the calorimetric signal and the thermostat temperature. In skin measurements, these steady-state variations typically reach 1 mV and 15 mK (peak-to-peak), resulting in a fluctuation of 4 mW (peak-to-peak) in the calculated *W*_*1*_ values.

### 4.2. Determination of skin thermal resistance and core temperature

As previously discussed, we are able to determine the skin heat flux and its heat capacity. Another variable of great interest that can be assessed is the thermal resistance of the skin (*R*_*skin*_), which we define as the ratio between the skin temperature change Δ*T*_*1*_, determined from the thermostat temperature and the calorimetric signal, and the skin heat flux change Δ*W*_*1*_:


$$\begin{array}{c} T_{1}=T_{2}+y/k\;\\ R_{skin}=\frac{\Delta T_{2}+\Delta y/k}{\Delta W_{1}}=\frac{\Delta T_{1}}{\Delta W_{1}} \end{array}$$ (8)

Once *R*_*skin*_ is known, it is possible to estimate the core body temperature at the location where the calorimeter is applied. This is done by extrapolating the linear temperature gradient across the skin, from the measured skin surface temperature *T*_*1*_ and the steady-state heat flux *W*_*1*_. Then, *T*_*core*_ is calculated by the following expression:


$$T_{core}=T_{2}+\frac{y}{k}+W_{1}\cdot R_{skin}=T_{1}+W_{1}\cdot R_{skin}\;$$ (9)

This approach shares conceptual similarities with the Zero Heat Flux (ZHF) method [35]. In that method, the estimation of core temperature relies on the assumption of steady-state, one-dimensional heat transfer across the skin. However, our method does not eliminate the heat flux, but instead uses its measured value to compute the local core temperature through the skin’s thermal resistance. This configuration, combined with an accurate quantification of heat losses (**Fig 6**), enables reliable measurements without full reliance on thermal insulation, and allows the full characterization of the heat transfer phenomena between the calorimeter and the skin.

### 4.3. Application measurements at rest

**Fig 8:**
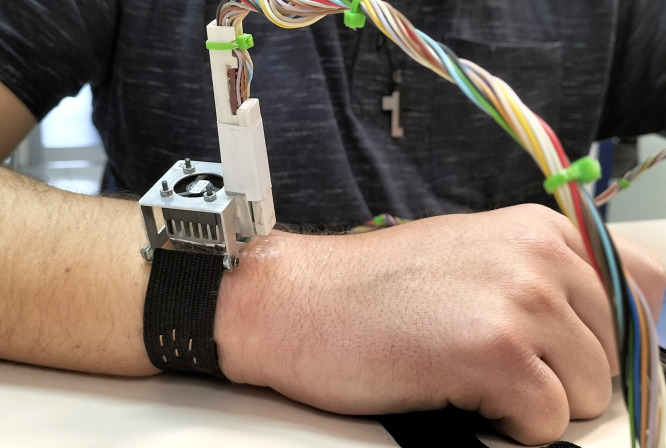
Calorimeter placed on the wrist. Application of the calorimeter on the wrist of a healthy 30-year-old male subject.

In this section, we present measurements performed on a healthy 30-year-old male subject, on the dorsal and volar areas of the wrist (see **Fig 8**), using a thermostat temperature step from 28 to 37°C (Δ*T*_*2 *_= 9°C). The subject was seated and at rest during the measurement. The magnitudes measured are the skin heat flux *W*_*1*_, its heat capacity and thermal resistance (*C*_*skin*_ and *R*_*skin*_), and the core temperature at the measurement site (*T*_*core*_).

**Fig 9:**
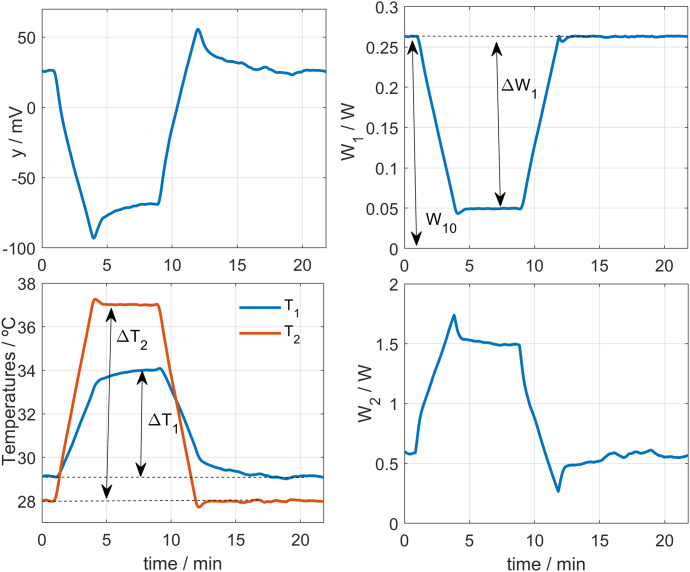
Measurement on the volar zone of the wrist. Measurement performed on the volar wrist area of a healthy 30-year-old male subject at rest, using the *S2* calorimeter, at a room temperature of 18.6°C and 49% relative humidity.

**Fig 9** shows one of the measurements performed on the wrist, specifically using calorimeter *S2* on the volar zone. The figure displays the experimental curves (*y*, *T*_*2*_ and *W*_*2*_) and the calculated ones: the temperature *T*_*1*_ and the heat flux *W*_*1*_, determined by Eq. 6. **Table 5** summarizes the results obtained for this and three additional measurements. Reported values include ambient temperature, relative humidity, heat flux *W*_*10*_ and its variation Δ*W*_*1*_, the thermal properties of the skin (*C*_*skin*_ and *R*_*skin*_), and the internal core body temperature *T*_*core*_.

**Table 5:** Thermal magnitudes on the wrist at rest.

Calorimeter	zone	*T*_*room*_	HR	Δ*T*_*1*_	*W*_*10*_	Δ*W*_*1*_	*C*_*skin*_	*R*_*skin*_	*T*_*core*_
*S1*	Dorsal^a^	19.6	53	5.4	0.144	0.175	2.3	30.8	32.2
*S2*	Dorsal^a^	19.6	53	5.3	0.109	0.181	2.4	29.4	30.6
*S1*	Volar	18.6	49	5.1	0.267	0.205	1.8	24.7	35.6
*S2*	Volar	18.6	49	4.9	0.264	0.214	1.8	22.7	35.1
		ºC	%	ºC	W	W	J/K	K/W	ºC

Results of measurements performed on the wrist of a healthy 30-year-old male subject at rest. Both calorimeters were used. The table includes the ambient conditions and the thermal parameters derived from the measurements.

^a^The subject experienced a sensation of cold in the measurement at dorsal zone.

The results shown in the **Table 5** correspond to a 4 cm² skin area. On the dorsal side of the wrist, the measured heat capacity was 2.3 J/K, with a thermal resistance of 33 ± 4 K/W. On the volar side, both values were lower: 1.8 J/K for heat capacity and 24 ± 2 K/W for thermal resistance. These parameters (*C*_*skin*_ and *R*_*skin*_) appear to remain stable regardless of variations in thermostat, ambient, or core body temperatures under resting conditions, which agrees with previous studies in some cases [17,19,20,24]. However, as discussed in the Introduction, physiological or environmental factors can also influence these values. **Table 5** presents two measurements with abnormally low core temperature, consistent with the subject’s reported sensation of cold. The higher value of the thermal resistance may indicate a slight local vasoconstriction.

### 4.4. Application measurements at exercise

**Fig 10:**
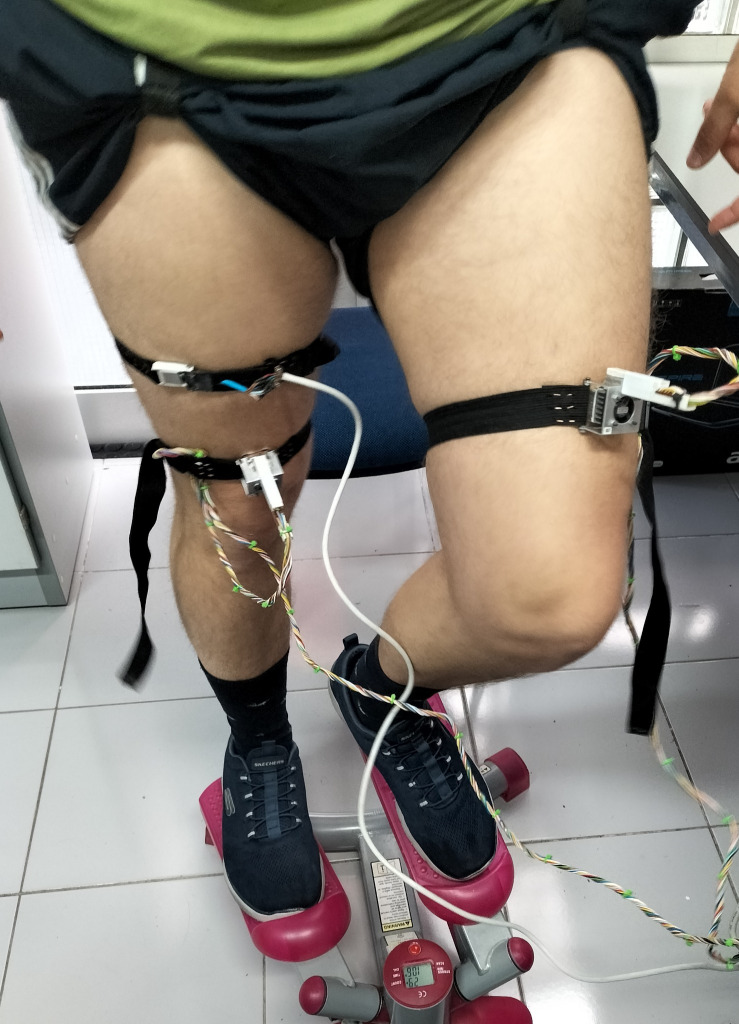
Measurement during exercise. A healthy 28-year-old male subject performing moderate physical activity on a stepper, while the thigh heat flux is measured by the calorimeters.

**Fig 11:**
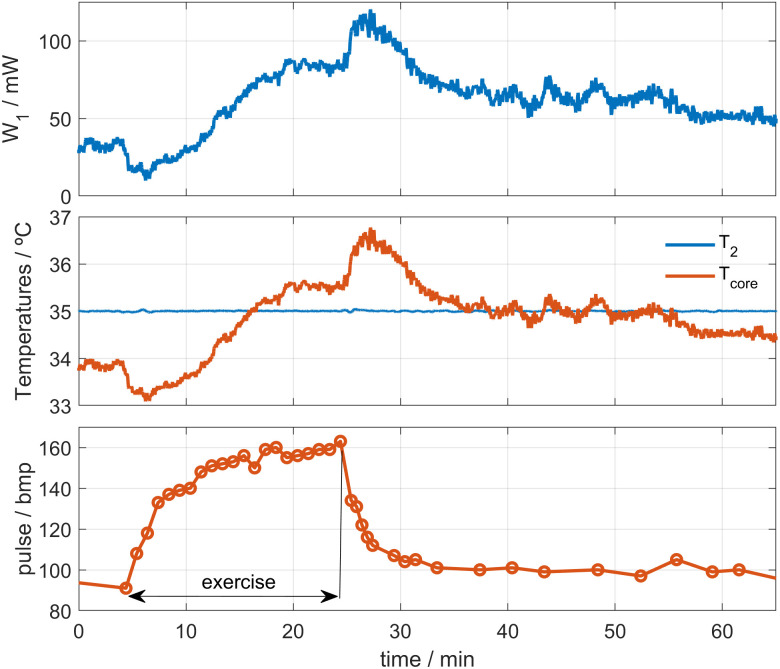
Heat flux, heart rate and core temperature during exercise. Heat flux *W*_*1*_, thermostat and core temperatures (*T*_*2*_ and *T*_*core*_) and the heart rate from the measurement performed during physical activity. The ambient temperature was 24.3°C.

To conclude the calorimeter applications, we present a heat flux measurement during moderate exercise on a stepper (**Fig 10**), on the thigh of a healthy 28-year-old male subject. The exercise was performed at a constant workload of 75 W, which corresponds to a moderate intensity for an untrained individual. Cadence was monitored by tracking the number of steps over time, and heart rate was recorded simultaneously. The exercise intensity and duration were chosen to avoid sweating, while ensuring a measurable signal. All measurements were performed in a controlled environment with 40–55% relative humidity. *W*_*1*_ was calculated using the first equation of the RC model (Eq. 2). *C*_*skin*_ and *R*_*skin*_ were determined in a previous measurement, obtaining *C*_*skin*_ = 3.8 J/K and *R*_*skin*_ = 26 K/W. The measurement was performed using the calorimeter *S1* at an ambient temperature of 24.3°C. The thermostat was set to *T*_*2*_ = 35°C, with a cooling current of *I*_*pel*_ = 50 mA. The average value of *T*_*0*_ was 0.52°C. **Fig 11** shows the measured skin heat flux *W*_*1*_, the thermostat and core temperatures, and the heart rate. As we can see, the heat flux decreases slightly during the first 7 min after the exercise begins. Once the activity stops, the heat flux shows an increase for approximately 6 minutes, and then gradually decreases over time. The core temperature, measured indirectly using Eq. 9, follows a similar behavior, as expected. This response is consistent with previous works [7–9], specifically in the thermal response shape [7].

## 5. Simulations

To complement the experimental application of the calorimeter, this section presents several simulations carried out with two main objectives. The first is to clearly define the operating range of the calorimeter by identifying the maximum and minimum programmable thermostat temperatures. The second is to understand how the calorimeter responds to changes in the thermal conditions of the skin when the core temperature of the tissue remains constant. This corresponds to a situation in which the subject is at rest.

The model’s differential equations were solved numerically using the finite difference method, with a time step equal to the experimental sampling period (Δt = 1 s). This approach facilitates implementation in the calorimeters acquisition and control program, written in C++. The maximum deviation between the simulated calorimetric signal and that obtained from the analytical expression is 0.2 mV, and the maximum deviation in the thermostat temperature is 0.01°C. Considering that the experimental noise of the calorimetric signal in steady state is ± 0.3 mV and the thermostat temperature fluctuates by ± 0.01°C, we consider the simulation results acceptable.

### 5.1. Operating range of the calorimeter

The operating range of the calorimeter is defined by the maximum and minimum thermostat temperatures that can be programmed. If the thermostat is unable to reach the set temperature, the system enters saturation. This occurs when the power required by the heating element falls outside its allowed limits (between 0 and 2 watts). The thermostat power *W*_*2*_ is regulated by a PID controller and depends on the power transferred to the calorimeter *W*_*1*_ and the ambient, cooling, and thermostat temperatures. If the required thermostat power is less than zero, the system is in lower saturation; if it exceeds the maximum allowed power, it is in upper saturation. In both cases, temperature control is lost. Using the calorimetric model (Eq. 2), we can simulate how the system behaves for different conditions. These simulations help to select the appropriate supply current for the cooling thermopile, ensuring that the thermostat remains within its operating range and saturation is avoided. From Eq. 2, the thermostat power can be isolated, resulting in the following expression:


$$W_{2}=\alpha_{1}\frac{dT_{2}^{2}}{dt^{2}}+\alpha_{2}\frac{dT_{2}}{dt}-\alpha_{3}\frac{dW_{2}}{dt}+\beta_{1}T_{2}-\beta_{2}W_{1}-\beta_{3}T_{01}-\beta_{4}T_{02}$$ (10)

…where the coefficients of the equation are given by:


$$\begin{array}{cccc} \alpha_{1}=\frac{C_{1}C_{2}}{P_{1}+P_{12}}\;\;\;\;\;\;\;\;\; & \alpha_{2}=C_{2}+C_{1}\frac{P_{2}+P_{12}}{P_{1}+P_{12}} & \alpha_{3}=\frac{C_{1}}{P_{1}+P_{12}}\\ \beta_{1}=P_{2}+\frac{P_{1}P_{12}}{P_{1}+P_{12}}\;\;\;\;\; & \beta_{2}=\frac{P_{12}}{P_{1}+P_{12}} & \beta_{3}=\frac{P_{1}P_{12}}{P_{1}+P_{12}}\;\;\;\;\; & \beta_{4}=P_{2} \end{array}$$ (11)

In the thermostat power equation (Eq. 10) there are both positive and negative terms. Increasing the rate of thermostat temperature variation (in K/min) requires more power. The ambient temperature (*T*_*01*_) and the cooling system temperature (*T*_*02*_) also influence the amount of power required to reach the set temperature. When a steady-state temperature is reached, the first three terms on the right-hand side of Eq. 10 become zero and do not contribute to the calculation of *W*_*2*_. This allows the determination of *W*_*2*_ for two different steady-state temperatures. Once these stationary power values are known, and considering the linear variation of the thermostat temperature, the first three terms, corresponding to the derivatives of *T*_*2*_ and *W*_*2*_, can be calculated.

S1 Fig shows three simulations of a Joule calibration measurement. The thermostat temperature varies from 28 to 37°C, with both heating and cooling phases performed at a rate of 3 K/min. The calibration base power is initially set to zero. After 5 minutes, it increases to 200 mW. Then, while the thermostat temperature rises, the heating power is reduced to 100 mW. Finally, when the thermostat cools back down to its initial temperature of 28°C, the heating power returns to 200 mW. This power profile (*W*_*1*_) corresponds to the blue curve shown.

In the first simulation (S1 Fig **A**), the room temperature is 20°C, *T*_*0*_ is estimated as 2°C (from Eq. 4) and the cooling thermopile current is *I*_*pel*_ = 50 mA. The simulated thermostat power *W*_*2*_ (in red) and the value computed using Eq. 10 (in green) are shown. Although Eq. 10 does not capture transient behavior accurately, it is useful for analytically determining the maximum and minimum *W*_*2*_ values, and for checking whether saturation occurs. In this first case, no saturation is observed, and the calorimeter operates correctly.

In the second simulation (S1 Fig B), the same procedure is followed, but with a room temperature of 28°C. In this case, lower saturation occurs. The value of *W*_*2*_ computed from Eq. 10 becomes negative, so the system fails to reach the set temperature. To solve this, in the third simulation (S1 Fig C), the cooling current is increased to *I*_*pel*_ = 150 mA. This adjustment allows the thermostat to reach the desired temperature without saturation.

In summary, the operating range depends on the ambient temperature, the heat flux *W*_*1*_ that passes through the calorimeter, the thermostat temperature setting and the cooling system temperature. If we consider a heat flux of *W*_*1*_ = 200 mW, a thermostat temperature variation from 28 to 37°C at a 3 K/min rate, and *T*_*0*_ = 2°C, the operating range of each calorimeter can be tabulated. S1 Table presents the maximum and minimum power required in the thermostat’s heating resistor, calculated using Eq. 10, for different ambient temperatures and different supply currents to the cooling thermopile, for each calorimeter. Zones where lower or upper saturation occurs are marked in red. For other operating conditions, a new table should be generated.

### 5.2. Thermal interaction calorimeter – skin

In this section, we simulate the operation of the calorimeter while varying the thermostat temperature, for different local core temperatures. When the calorimeter is applied on the skin, the transmitted power *W*_*1*_ from the skin to the calorimeter is given by:


$$W_{1}=(T_{core}-T_{1})\cdot P_{skin}\;$$ (12)

…where *T*_*core*_ is the core temperature in the area where the measurement is performed, *T*_*1*_ is the skin temperature (which also corresponds to the temperature of the first calorimetric model domain) and *P*_*skin*_ is the thermal conductance of the skin (*P*_*skin*_ = 1/*R*_*skin*_). If we substitute Eq. 12 in the calorimetric model (Eq. 2), we obtain:


$$\begin{array}{c} T_{core}P_{skin}=\frac{C_{1}}{k}\frac{dy}{dt}+\frac{P_{1}+P_{12}+P_{skin}}{k}y+C_{1}\frac{dT_{2}}{dt}+(P_{1}+P_{skin})T_{2}-P_{1}T_{01}\;\\ W_{2}=-\frac{P_{12}}{k}y+C_{2}\frac{dT_{2}}{dt}+P_{2}(T_{2}-T_{02}) \end{array}$$ (13)

As previously discussed, **Fig 6** shows a diagram illustrating the direction of the heat flux *W*_*1*_ leaving the human body. This heat flux passes through the calorimeter and splits into two: the heat flux through the thermopile *W*_*12*_ and the portion transmitted to the environment *W*_*10*_. Thus, we have:


$$\begin{array}{c} W_{12}=P_{12}(T_{1}-T_{2})=P_{12}y/k\;\\ W_{10}=P_{1}(T_{1}-T_{10})=P_{1}(T_{2}+y/k)-P_{1}(T_{room}+T_{0}+\alpha I_{pel}) \end{array}$$ (14)

When the calorimeter is applied on the skin, a thermostat temperature variation produces a change in the temperature *T*_*1*_ and consequently, in the calorimetric signal. Analyzing the response to this excitation allows the determination of *C*_*1*_ (*C*_*0*_ + *C*_*skin*_), *P*_*skin*_ and *T*_*core*_ (Eq. 8 & Eq. 9). In Section 4.3 we presented the results of measurements performed on the dorsal and volar areas of the wrist. In these measurements (**Table 5**) it has been found that *T*_*core*_ is different in each measurement, but practically constant during each individual measurement.

For the simulations presented in this section, we assume *T*_*core*_ = 35°C and *R*_*skin*_ = 25 K/W, which correspond to a thermal conductance *P*_*skin*_ = 0.04 W/K. Two different ambient temperatures are considered: 20°C and 25°C. The thermostat temperature is varied from 28°C to 38°C, and the cooling current is 100 mA. Under these conditions, the operation of the calorimeter is simulated with Eq. 13. S2 Fig shows the results of the simulations.

S2 Table shows a summary of the numerical values obtained from the simulation and incorporates a brief discussion about the experimental uncertainty propagation. As shown, the induced variations of skin temperature and heat flux (Δ*T*_*1*_ and Δ*W*_*1*_) are independent of the ambient temperature. The variation of *T*_*core*_ produces variations in both *T*_*1*_ and *W*_*1*_, but the ratio Δ*T*_*1*_/ Δ*W*_*1*_ remains constant. Furthermore, this ratio in unaffected by the thermostat temperature settings. This means that any variations in *R*_*skin*_ are not directly related to the temperatures changes, but rather to their effects that on the skin, or to other physiological mechanisms such as vasodilatation or vasoconstriction. An important outcome of this simulation is the shape of the signal *W*_*1*_ in response to changes in thermostat temperature. In Eq. 10, we proposed the hypothesis that *W*_*1*_ would follow the same shape as the thermostat temperature function, but with the sign changed. This hypothesis is confirmed by the simulation results.

## 6. Discussion

This work shows the current stage of development and use of the skin calorimeters. These devices have been used to study heat flux and thermal properties of the skin at rest, for a wide range of temperatures and environmental conditions, with an accuracy is ± 2 mW for heat flux, ± 1 K/W for thermal resistance, and ±0.05 J/K for heat capacity, for a 2 × 2 cm² skin area. For example, the skin calorimeters have proven useful to monitor the evolution of second-degree skin burns [5] or characterizing anatomical differences [34].

With this work, we have now started to studying applications during physical exercise. We have shown that it is possible to monitor heat flux and estimate core temperature in localized skin areas, such as specific muscles (in this case, the rectus femoris), during moderate physical activity. This opens a new field of monitoring that complements thermographic measurements. However, this application has some limitations related with sweating, which we are currently studying. Including only one subject in the exercise experiment is another limitation of this study. We are currently performing additional experiments with a larger sample.

## 7. Conclusions

This study presents the development and application of a wearable, non-differential skin calorimeter, able of measuring the thermal magnitudes of the human skin *in vivo,* of a localized area of 2 × 2 cm^2^:

- The calorimetric model developed was successfully applied in three experimental conditions: electrical calibrations, *in vivo* skin measurements, and empty tests. In all cases, the model showed good agreement with the measured signals.
- The incorporation of the skin into the calorimetric model enables the estimation of the core temperature at the measurement location. This estimation is based on the measured heat flux and skin thermal resistance, without requiring heat flux suppression as in Zero Heat Flux (ZHF) methods, and without full reliance on thermal insulation.
- The instrument and its operating model allows the determination of skin thermal magnitudes of great interest under resting conditions: surface temperature, heat flux, heat capacity, thermal resistance, and core temperature. The device is also able to measure the heat flux during moderate physical activity, and it is capable of estimating the core temperature in such conditions.
- Numerical simulations based on the calorimetric model were used to determine the operating range of the device. The simulations identified the conditions in which the thermostat can reach its programmed temperature, avoiding saturation. Additional simulations revealed that the equivalent thermal resistance of the skin depends primarily on the physiological state of the subject. It remains independent of both ambient temperature and thermostat settings, unless these conditions induce physiological alterations.

This wearable calorimeter shows potential as a non-invasive tool for assessing local skin thermal properties under both rest and exercise conditions. Its compact format may allow integration into multisensor platforms for physiological monitoring, although further testing is needed to explore its full applicability.

## Supporting information

S1 Fig Simulation results for a Joule calibration for *T*_*room*_ = 20°C (A) and *T*_*room*_ = 28°C (B and C). Thermostat temperature programmed from 28°C to 37°C in all cases.
(TIF)

S2 Fig Simulation of the calorimeter applied on the skin, for an ambient temperature of *T*_*room*_ = 20°C (A), and 25°C (B). (TIF)

S1 Table Operating range of the calorimeters. (DOCX)

S2 Table Results of the simulation of the calorimeter – skin interaction. (DOCX)

S1 Data Files Calibration, rest and exercise measurement samples.
(ZIP)
