# Supplementary figures and images for: Development & application of a wearable non-differential calorimeter for skin heat transfer analysis
Source: PLoS One. 2025 Oct 17;20(10):e0334062. doi: 10.1371/journal.pone.0334062 (PMC12533852; doi:10.1371/journal.pone.0334062)

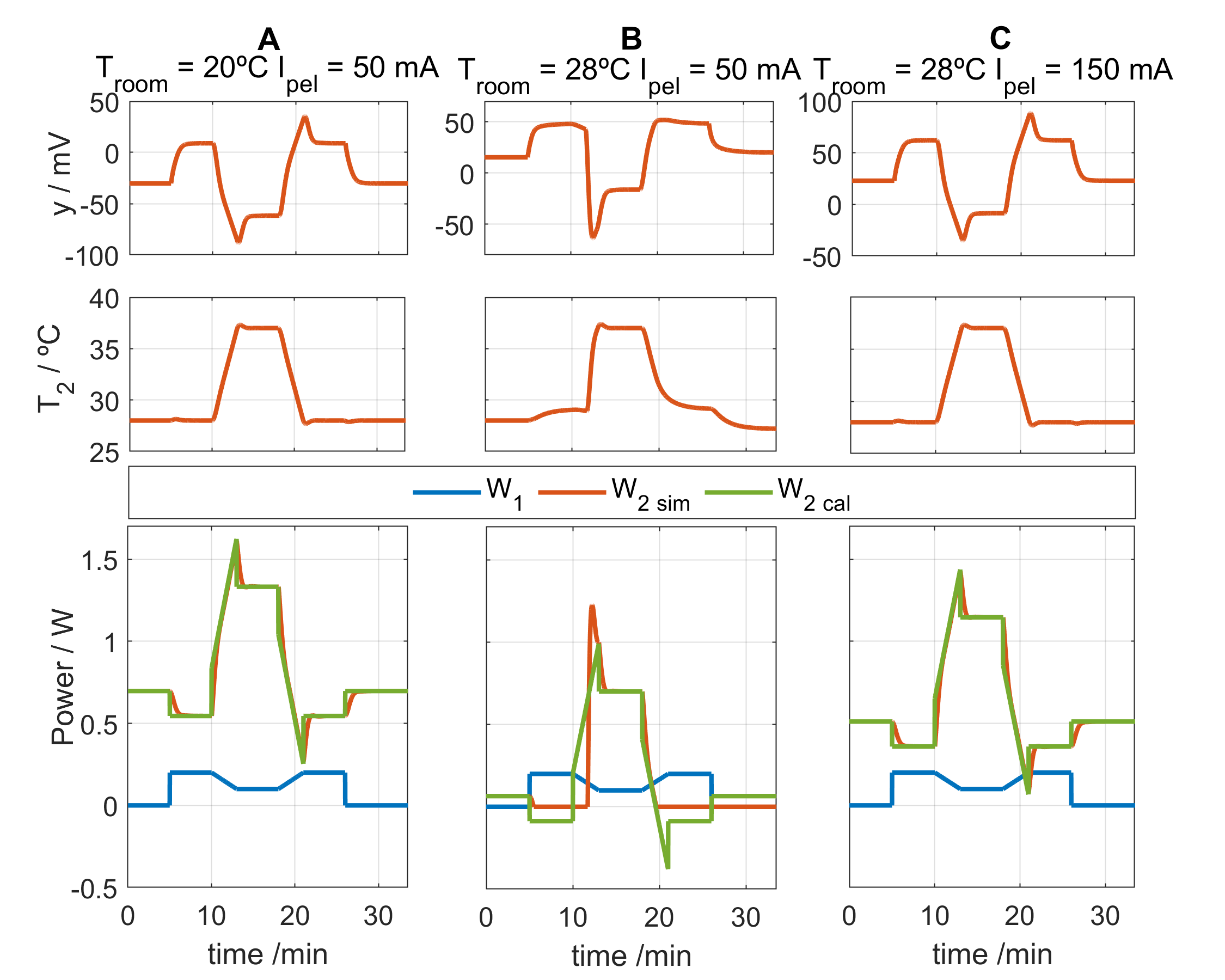

Supplement: S1 Fig — Thermostat temperature programmed from 28°C to 37°C in all cases. (TIF) [file pone.0334062.s001.tif]

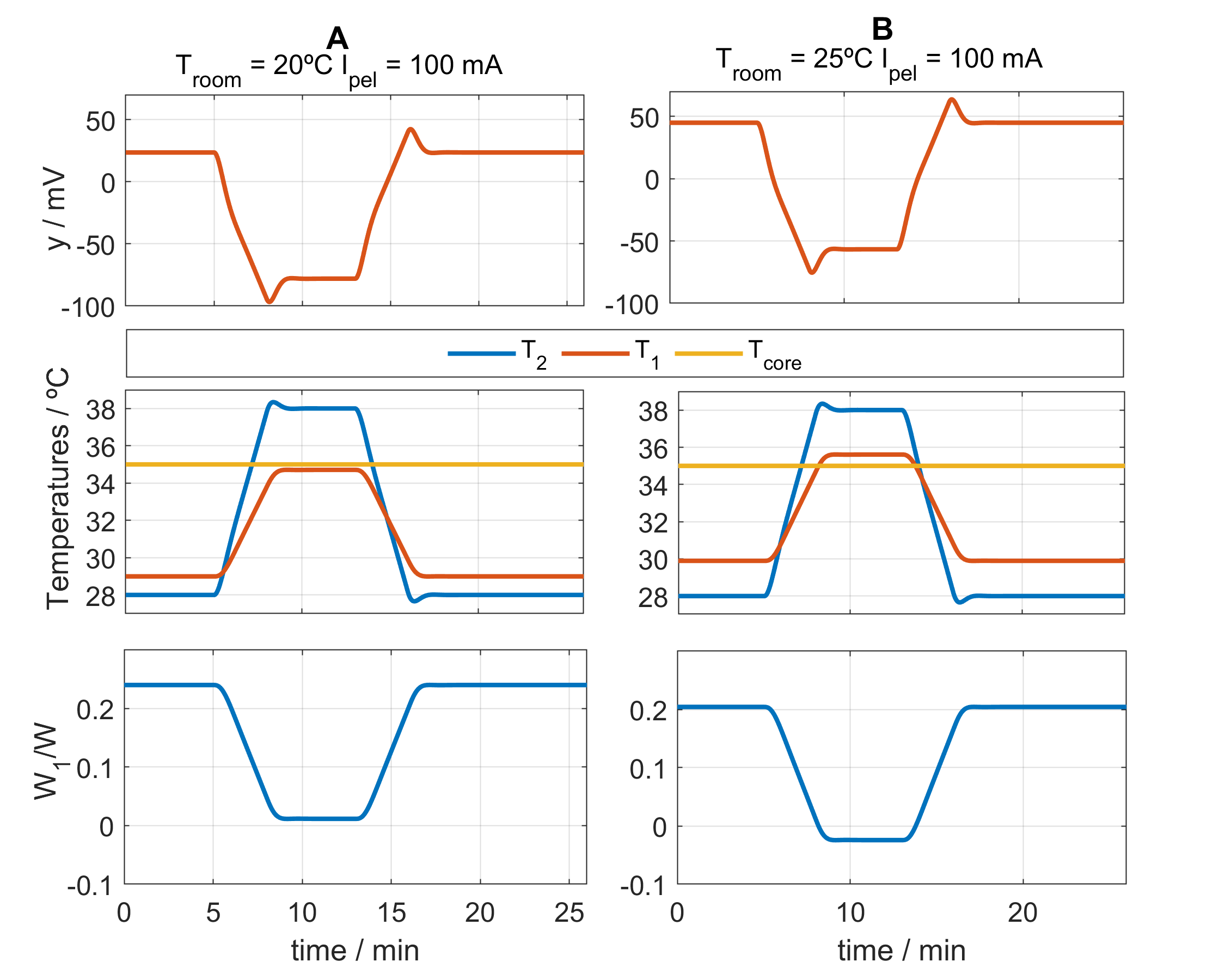

Supplement: S2 Fig — (TIF) [file pone.0334062.s002.tif]
