# Supplementary material for: Development & application of a wearable non-differential calorimeter for skin heat transfer analysis
Source: PLoS One. 2025 Oct 17;20(10):e0334062. doi: 10.1371/journal.pone.0334062 (PMC12533852; doi:10.1371/journal.pone.0334062)
Supplement: S1 Table — (DOCX) [file pone.0334062.s003.docx]

**S1 Table.** Operating range of the calorimeters.

| *S1* skin calorimeter | | | | | | |
| --- | --- | --- | --- | --- | --- | --- |
| *I_pel_* / mA | *T_room_* = 18 ºC | *T_room_* = 20 ºC | *T_room_* = 22 ºC | *T_room_* = 24 ºC | *T_room_* = 26 ºC | *T_room_* = 28 ºC |
| 50 | 1845 426 | 1686 268 | 1528 109 | 1370 -49 | 1212 -207 | 1054 -365 |
| 100 | 2067 649 | 1909 491 | 1751 332 | 1593 174 | 1435 16 | 1277 -142 |
| 150 | 2290 872 | 2132 713 | 1974 555 | 1816 397 | 1658 239 | 1500 81 |
| 200 | 2513 1095 | 2355 936 | 2197 778 | 2039 620 | 1881 462 | 1723 304 |
| *S2* skin calorimeter | | | | | | |
| *I_pel_* / mA | *T_room_* = 18 ºC | *T_room_* = 20 ºC | *T_room_* = 22 ºC | *T_room_* = 24 ºC | *T_room_* = 26 ºC | *T_room_* = 28 ºC |
| 50 | 1789 405 | 1635 251 | 1481 98 | 1327 -56 | 1174 -210 | 1020 -364 |
| 100 | 2000 617 | 1846 463 | 1693 309 | 1539 155 | 1385 2 | 1231 -152 |
| 150 | 2211 828 | 2058 674 | 1904 521 | 1750 367 | 1596 213 | 1443 59 |
| 200 | 2423 1039 | 2269 886 | 2115 732 | 1962 578 | 1808 424 | 1654 271 |

Maximum and minimum power values (in mW) that must be dissipated by the thermostat (*W_2_*​) as a function of the cooling thermopile current (*I_pel_*​) and ambient temperature (*T_room_*​), in order to control the thermostat temperature between 28 and 37 °C at a 3 K/min rate. A heat flux of *W_1_* = 200 mW is assumed. Regions where saturation occurs are marked in red.
