# Supplementary material for: Development & application of a wearable non-differential calorimeter for skin heat transfer analysis
Source: PLoS One. 2025 Oct 17;20(10):e0334062. doi: 10.1371/journal.pone.0334062 (PMC12533852; doi:10.1371/journal.pone.0334062)
Supplement: S2 Table — (DOCX) [file pone.0334062.s004.docx]

**S2 Table.** Results of the simulation of the calorimeter – skin interaction.

***A) T_core_* variation**

| *T_room_* / ºC | *T_2_* / ºC | *C_1_* / J/K | *T_core_* / ºC | *T_10_*/ ºC | *T_1_* / ºC | *W_10_* mW | *W_1_* / mW | *R_skin_* / KW^-1^ |
| --- | --- | --- | --- | --- | --- | --- | --- | --- |
| 20 | 28 to 38 | 4.0 | 34.5 | 28.86 | 34.59 | 225.5 | -3.6 | 25.0 |
| 20 | 28 to 38 | 4.0 | 35.0 | 28.99 | 34.71 | 240.5 | 11.4 | 25.0 |
| 20 | 28 to 38 | 4.0 | 35.5 | 29.11 | 34.84 | 255.6 | 26.4 | 25.0 |
| 25 | 28 to 38 | 4.0 | 34.5 | 29.76 | 35.49 | 189.5 | -39.6 | 25.0 |
| 25 | 28 to 38 | 4.0 | 35.0 | 29.89 | 35.62 | 204.5 | -24.6 | 25.0 |
| 25 | 28 to 38 | 4.0 | 35.5 | 30.01 | 35.74 | 219.5 | -9.6 | 25.0 |

Simulated values of skin temperature variation (*T_10_ and T_1_*) and heat flux variation (*W_10_ and W_1_*) for *R_skin_* = 25 K/W and different core and ambient temperatures (*T_core_* and *T_room_*). The skin thermal resistance is calculated as *R_skin_* = Δ*T_1_*/Δ*W_1_*. Fig. S3 shows the case for *T_core_* = 35 ºC, where *T_2_* varies linearly from 28 to 38 ºC over a period of 3 min.

***B) R_skin_* variation**

| *T_room_* / ºC | *T_2_* / ºC | *C_1_* / J/K | *T_core_* / ºC | *T_10_* / ºC | *T_1_* / ºC | *W_10_* / mW | *W_1_*/ mW | *R_skin_* / KW^-1^ |
| --- | --- | --- | --- | --- | --- | --- | --- | --- |
| 20 | 28 to 38 | 4.0 | 35.0 | 29.34 | 34.73 | 283.3 | 13.4 | 20.0 |
| 20 | 28 to 38 | 4.0 | 35.0 | 28.99 | 34.71 | 240.5 | 11.4 | 25.0 |
| 20 | 28 to 38 | 4.0 | 35.0 | 28.73 | 34.70 | 209.1 | 9.9 | 30.0 |
| 25 | 28 to 38 | 4.0 | 35.0 | 30.19 | 35.58 | 240.7 | -29.0 | 20.0 |
| 25 | 28 to 38 | 4.0 | 35.0 | 29.89 | 35.62 | 204.5 | -24.6 | 25.0 |
| 25 | 28 to 38 | 4.0 | 35.0 | 29.67 | 35.64 | 177.8 | -21.4 | 30.0 |

Simulated values of skin temperature variation (*T_10_ and T_1_*) and heat flux variation (*W_10_ and W_1_*) for *T_core_* = 35 ºC and different *R_skin_* and ambient temperature *T_room_*. The skin thermal resistance is calculated as *R_skin_* = Δ*T_1_*/Δ*W_1_*. Fig. S3 shows the case for *T_core_* = 35 ºC, where *T_2_* varies linearly from 28 to 38 ºC over a period of 3 min.

**NOTE: EXPERIMENTAL UNCERTAINTY PROPAGATION**

The simulations performed do not account for the uncertainty of the model parameters listed in Table 1. Considering the oscillations of the calorimetric signal and the thermostat temperature, it is possible to estimate partial uncertainties and the resulting maximum uncertainty in the heat flux. For a calorimetric signal increase of 40 mV, a heat flux of 204 ± 5 mW is obtained, corresponding to an approximate uncertainty of 2.5% in the determination of skin heat flux. Regarding the uncertainty of skin thermal resistance, this depends directly on the thermostat temperature step. For a temperature step of 10 K, the uncertainty is 0.04 K/W, whereas for a step of 4 K the uncertainty increases to 0.11 K/W.
