# Supplementary material for: Development & application of a wearable non-differential calorimeter for skin heat transfer analysis
Source: PLoS One. 2025 Oct 17;20(10):e0334062. doi: 10.1371/journal.pone.0334062 (PMC12533852; doi:10.1371/journal.pone.0334062)
Supplement: S1 Data Files — Calibration, rest and exercise measurement samples. (ZIP) [file pone.0334062.s005.zip › Files.docx]

The dataset consists of four experimental files in .dat format:

1. **File1.dat**: Calibration measurements for several Peltier module input currents.
2. **File2.dat**: Joule calibration measurements at air for several Peltier module input currents.
3. **File3.dat**: Experimental measurement on the dorsal side of the left wrist of a 30-year-old subject.
4. **File4.dat**: Experimental measurement on the rectus femoris of a 28-year-old subject during moderate exercise on a stepper at 75 W.

Each file contains 7 columns with the following structure:

| **Column** | **Description** |
| --- | --- |
| 1 | Time (s) |
| 2 | Peltier module current *I_pel_* (A) |
| 3 | Calorimetric signal (mV) from the thermopile |
| 4 | Thermostat power (W) applied to a 6.40 Ω constantan resistance |
| 5 | Joule power applied to the measurement plate (W), 7.84 Ω constantan resistance (zero if at ir, or on skin) |
| 6 | Thermostat temperature (°C), measured with Pt100 sensor |
| 7 | Ambient temperature (°C), measured with a 5 kΩ thermistor |
